# Has the time come to change the treatment criteria for patients with chronic kidney disease? The “hypofiltering nephron” hypothesis

**DOI:** 10.3389/fneph.2025.1713215

**Published:** 2025-12-19

**Authors:** Giulio Romano, Gianfranco Ferraccioli, GianLuca Colussi

**Affiliations:** 1 Nephrology, Department of Medicine, University of Udine, Udine, Italy; 2 Department of Internal Medicine, Catholic University of the Sacred Heart, Rome, Italy; 3 Clinical Medicine, Department of Medical Sciences, University of Ferrara, Ferrara, Italy

**Keywords:** nephron, glomerular filtration rate, renal resistive index, atherosclerotic nephropathy, renal circulation, pentoxifylline

“Personalized medicine” denotes the future of disease treatment. This implies that etiologically distinct entities, even if they converge on a common disease pathway, may benefit from differentiated treatments. Chronic kidney disease (CKD) is one example of this, as it has long been regarded as a sort of common container in which patients are treated similarly regardless of the underlying disease. In fact, all patients with CKD are treated as if they meet Bricker’s “intact nephron” hypothesis (1). This hypothesis, which was developed in the 1960s, assumes that the residual nephrons compensate for the reduction in the number of functioning nephrons because of kidney injury by increasing the fractional contribution of each nephron to the total excretion rate. This mechanism reduces the decline in total kidney function by causing a single intact nephron to hyperfilter, which occurs by increasing the glomerular blood hydrostatic pressure (↑ΔP) in conjunction with its hypertrophy (2).

The current Kidney Disease: Improving Global Outcomes (KDIGO) CKD classification is based solely on estimated glomerular filtration rate (GFR) and albuminuria categories and does not distinguish between different patterns of nephron function (3). As a result, mechanistically heterogeneous patients are grouped into the same prognostic category despite potentially distinct pathophysiological processes. The hyperfiltering nephron model has significantly influenced our understanding of CKD progression for decades. However, it fails to account for patients who experience a decline in GFR without the development of albuminuria, even in the presence of substantial vascular disease. To address this gap, we define a “hypofiltering nephron” as a structurally viable nephron with preserved filtration surface area but reduced single-nephron GFR (SNGFR) caused by increased preglomerular vascular resistance and hypoperfusion. This model complements, rather than contradicts, Bricker’s paradigm and may help clarify a significant and poorly understood group of nonalbuminuric CKD patients.

**Figure 1:**
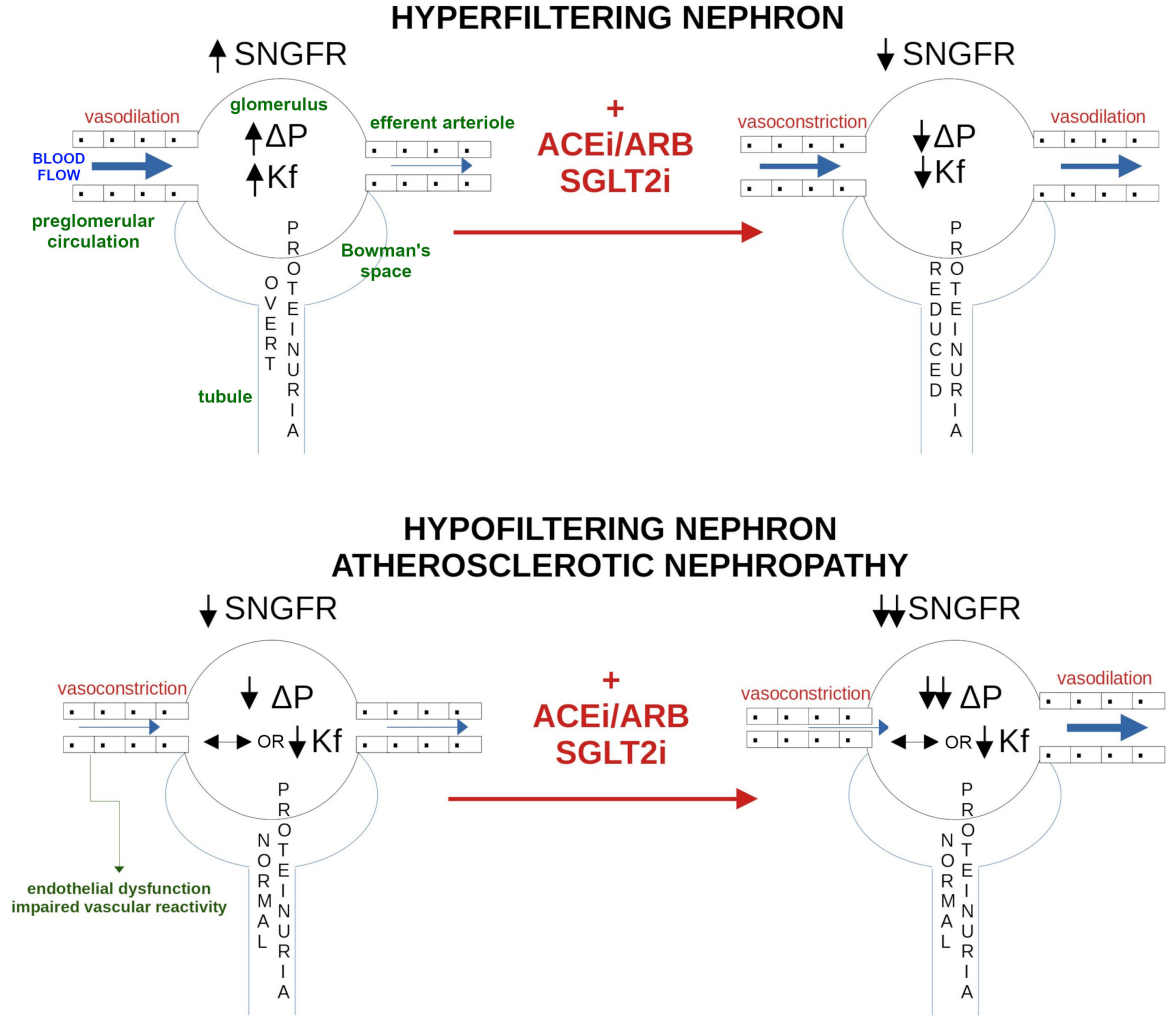
Residual nephrons increase their fractional contribution to global filtration rate to compensate for the decrease in nephron number caused by glomerular injury. Hyperfiltration and residual nephron hypertrophy increase glomerular blood hydrostatic pressure (↑ΔP), preventing kidney function decline. In the long term, this compensatory process raises glomerular basal membrane permeability (↑Kf), resulting in overt albuminuria. This pathophysiology suggests that interventions aimed at reducing nephron hyperfiltration and albuminuria may prevent kidney disease progression. The same treatment, however, may not be appropriate in nonalbuminuric atherosclerotic nephropathy, in which the increased resistance of medium-sized intrarenal arteries hypoperfuses the nephrons, lowering SNGFR without overt albuminuria. Ang II-induced efferent vasoconstriction limits the decline in SNGFR. If Ang II is inhibited (ACEi/ARB/SGLT2i) or afferent vasoconstriction is enhanced (SGLT2i), SNGFR and overall kidney function might be compromised. SNGFR, single nephron glomerular filtration rate; Ang-II, angiotensin II; ACEi, angiotensin-converting enzyme inhibitors; ARB, angiotensin receptor blockers; SGLT2i, sodium-glucose cotransporter 2 inhibitors.

Hyperfiltration models suggest that increased ΔP, seen in conditions such as hypertension-related nephropathy, reduced kidney mass nephrectomy, albuminuric diabetes, or other CKD secondary to glomerulonephritis evolution, including obesity-related glomerulopathy, results in mechanical stress on glomerular capillaries (4, 5). This mechanical stress activates complex molecular pathways that facilitate the elongation of capillaries and expand their surface area as a compensatory mechanism, ultimately leading to increased collagen synthesis and fibrosis (6). Mechanical stress, together with inflammatory and oxidative signals, promotes podocyte injury and reduces podocyte density, compromising the filtration barrier. The resulting increase in the ultrafiltration coefficient (Kf), reflecting a larger effective filtration surface and higher permeability, leads to albuminuria, which further accelerates sclerosis in residual nephrons (7). This pathogenesis justifies the use of treatments that lower glomerular hyperfiltration and reduce protein loss in the urine, which can slow the progression of kidney disease (see **Figure 1**).

The “hypofiltering nephron” model proposes that vascular and tubulointerstitial dysfunction can occur without glomerular hyperfiltration (8). A hypofiltering nephron remains structurally intact but is underperfused due to increased resistance before the glomerulus, resulting in a reduced SNGFR despite a preserved filtration surface area. This situation is distinct from (i) nephron loss, where filtration units are completely absent, and (ii) the failure of compensatory hyperfiltration in the remaining nephrons, as described by Bricker. Although these mechanisms may coexist throughout the progression of nonalbuminuric CKD, only the first mechanism truly reflects vascular-driven hypofiltration. A clear distinction between the two models is reported in **Table 1**.

**Table 1:** Principal features of the two models.

Feature	Hyperfiltering nephron	Hypofiltering nephron
Primary driver	↑ΔP, ↑Kf, glomerular hyperperfusion	↑medium-sized intrarenal artery resistance, ↓renal plasma flow
Albuminuria	present, often progressive	absent or minimal
SNGFR	increased	reduced
Residual nephron stress	hemodynamic & structural (barrier injury/remodeling)	ischemic/hypoxic & metabolic
Typical phenotype	albuminuric DN, obesity-related glomerulopathy, glomerulonephritis	aging kidney, ischemic or atherosclerotic nephropathy, nonalbuminuric DN

ΔP: glomerular blood hydrostatic pressure; Kf: ultrafiltration coefficient; SNGFR: single nephron glomerular filtration rate; DN: diabetic nephropathy. See text for explanation.

Accelerated vascular aging or systemic atherosclerosis can affect medium-sized intrarenal vessels, including interlobar and arcuate arteries. The increased resistance in these vessels leads to a reduced renal plasma flow and decreased total and single-nephron ultrafiltration coefficients (↓Kf), while the effect on glomerular capillary pressure (ΔP) remains uncertain in humans (9). This process progresses without the onset of pathological albuminuria due to the absence of glomerular hyperfiltration in residual nephrons and defines “atherosclerotic nephropathy”. In the aging as well as hypertensive kidney, nephrosclerosis is the most common finding, consisting of glomerulosclerosis, arteriosclerosis, tubular atrophy, and tubulointerstitial fibrosis (10). In some instances of aging kidneys, the anticipated residual glomerular hypertrophy and hyperfiltering compensation do not develop to counteract the decrease in GFR as nephrosclerosis progresses (10). Additionally, in cases of hypertension, arterial remodeling and atherosclerotic vascular dysfunction can lead to secondary chronic kidney ischemia and a further decline in GFR, even when there is no significant renal artery stenosis present (11). Moreover, diabetic nephropathy, especially when associated with older age or cardiovascular risk factors, can progress without signs of hyperfiltration or albuminuria (12). In our hypothesis, similar processes could explain the nonalbuminuric GFR decline and the evolution of “atherosclerotic nephropathy” (13), in which nephron hypoperfusion and failure to mount a hyperfiltration response may coexist and participate in nephron loss and CKD progression.

Aging, hypertension and diabetes are major risk factors for atherosclerotic cardiovascular disease and share a common background of chronic oxidative stress that reduces nitric oxide bioavailability and disrupts endothelial-dependent vasodilation (14). The resulting predominance of vasoconstrictor pathways, including endothelin-1 (ET-1) and angiotensin (Ang) II, promotes structural remodeling of the intrarenal vasculature, characterized by medial thickening, hyalinization and increased stiffness (15). These changes narrow the lumen of interlobar and arcuate arteries, reducing renal plasma flow and glomerular perfusion pressure. In residual nephrons, this manifests as a reduction in SNGFR without increased permeability of the glomerular barrier. Progressive hypoperfusion also contributes to chronic hypoxia, which accelerates tubulointerstitial fibrosis and nephron loss (16).

Atherosclerotic nephropathy is characterized by increased resistance in medium-sized preglomerular intrarenal arteries, which reduces renal plasma flow and leads to underperfusion of otherwise nonsclerotic nephrons, resulting in a hypofiltering, typically nonalbuminuric phenotype. Mechanistically, Carrara et al. found that in type 2 diabetes with hypertension and without albuminuria, a higher preglomerular/postglomerular resistance ratio was associated with a greater decline in GFR, indicating the significance of preglomerular vascular resistance and hypoperfusion in disease progression (17). Clinically, the intrarenal Doppler-derived renal resistive index (RRI) serves as an effective indicator of nephrovascular hemodynamics. It increases with age and correlates with CKD progression and fibrosis, independent of albuminuria (18, 19). In our retrospective cohort of high cardiovascular-risk patients with nonproteinuric CKD, an elevated RRI was linked to an accelerated decline in GFR over five years and increased long-term mortality (13). Additionally, the most significant decline in GFR was noted when proteinuria was present alongside elevated RRI (20). These observations support the hypofiltering-nephron concept: increased intrarenal vascular resistance correlates with CKD progression regardless of albuminuria, and its association with proteinuria may indicate a more severe, dual-mechanism pathway (hypoperfusion and barrier injury). Future mechanistic studies are required to evaluate causality and therapeutic implications.

We believe that the evidence available today strongly suggests that patients with CKD and hypofiltering residual nephrons should be treated differently than those with hyperfiltration of residual nephrons, because in the former case, it is precisely the generalized hypofiltration that causes the GFR drop, and, in this case, drugs that exacerbate glomerular hypofiltration might worsen the kidney function. As a result, using the precision medicine principle, we should identify patients with nonalbuminuric CKD, atherosclerotic vascular disease, or increased RRI who could benefit from a tailored therapeutic approach based on the hypofiltering nephron hypothesis. The patient with progressive diabetic nephropathy is the best example. The development of Kimmelstiel-Wilson glomerulonephritis causes residual nephrons to become hyperfiltering and albuminuric in the albuminuric variant of diabetic nephropathy (21). However, nonalbuminuric diabetic nephropathy can also progress to significant CKD. Since glomerular blood flow is decreased because of the medium-sized intrarenal arteries’ atherosclerotic disease, the remaining nephrons in this latter scenario are hypofiltering, and albuminuria remains within the normal range. Garofolo et al. found that in patients affected by type 2 diabetes, those nonalbuminuric with CKD had a higher RRI than those nonalbuminuric without CKD. Additionally, nonalbuminuric patients with CKD had a lower kidney volume than those with albuminuric CKD. According to this study, patients with nonalbuminuric diabetic nephropathy may exhibit an atherosclerotic nephropathy since their RRI is increased and their kidney morphology suggests more advanced ischemic and fibrotic processes than those with albuminuric diabetic nephropathy (22).

Angiotensin-converting enzyme inhibitors (ACEi) and angiotensin receptor blockers (ARB) have not shown consistent kidney-protective effects in patients with nonalbuminuric CKD, regardless of diabetes status (23). Similarly, in the EMPA-KIDNEY trial, the sodium-glucose cotransporter 2 inhibitor (SGLT2i) empagliflozin slowed chronic GFR decline across all albuminuria categories, although the reduction in the primary composite outcome of kidney disease progression or cardiovascular death was not statistically significant in the strictly normoalbuminuric subgroup (24). Despite this, ACEi, ARB and SGLT2i remain the standard first-line therapy in diabetic nephropathy. This approach is appropriate for albuminuric nephropathy, where glomerular hyperfiltration and increased permeability of the filtration barrier accelerate sclerosis of the residual nephrons through Ang II–mediated mechanisms (25). In contrast, the same therapeutic strategy may be less appropriate in nonalbuminuric nephropathy. In this phenotype, viable nephrons are hypoperfused due to increased resistance in medium-sized intrarenal arteries; in selected hypofiltration phenotypes, suppressing Ang II may be counterproductive because Ang II–mediated efferent arteriolar constriction helps maintain glomerular pressure and partially preserve SNGFR. Consequently, treatments that reduce Ang II activity (ACEi or ARB) or further constrict the afferent arteriole (SGLT2i) (26) could compromise a compensatory mechanism that supports filtration in the hypofiltering nephron (see **Figure 1**).

In prospective, cautious, individualized use of ACEi, ARB, and SGLT2i may be appropriate in patients with nonalbuminuric CKD attributable to atherosclerotic nephropathy, given the possibility that further reductions in intraglomerular pressure could exacerbate hypofiltration in some patients. Agents that restore intrarenal perfusion or counter hypofiltration may offer greater benefit in this phenotype, but clinical evidence remains limited. We previously explored ET-1 pathway modulation because ET-1 is a potent vasoconstrictor in the intrarenal circulation. In patients with systemic sclerosis complicated by digital ulcers, without albuminuria or kidney failure but with elevated RRI, the dual ET-1 receptor antagonist bosentan did not change RRI but reduced mean arterial pressure and GFR (27). This pattern suggests that, in early atherosclerotic nephropathy with preserved or mildly reduced kidney function, ET-1 supports glomerular filtration and its blockade can impair the kidney function. These observations argue for cautious use of ET-1 antagonism in suspected nephrons hypofiltration until targeted trials are available. By contrast, pentoxifylline, a nonselective phosphodiesterase inhibitor with anti-inflammatory, antiproliferative, and antifibrotic properties, has been associated with less progression of subclinical atherosclerosis and a smaller GFR decline than controls in patients with diabetic nephropathy over 18 months (28). Although no direct trials have evaluated pentoxifylline specifically in nonalbuminuric CKD, its vascular and anti-inflammatory actions make it a hypothesis-generating option that warrants prospective evaluation in this population.

Although effective therapeutic strategies have been developed to prevent CKD in patients with albuminuria, specific strategies for those without albuminuria and increased intrarenal vascular resistance remain lacking. Considering that patients affected by nonalbuminuric CKD outnumber those with albuminuria (29), it is essential to enhance our understanding of this predominant form of CKD progression to develop new tailored therapies. In clinical settings, a combined biomarker–imaging approach may be the best strategy. This can include measurements of circulating markers of endothelial dysfunction or injury that have been correlated with kidney outcomes such as ET-1, asymmetric dimethylarginine (ADMA), soluble vascular or intracellular adhesion molecules (VCAM-1 or ICAM-1), von Willebrand factor (vWF), and high-sensitivity C-reactive protein (hs-CRP) (30). Additionally, imaging modalities such as Doppler-derived RRI, contrast-enhanced ultrasonography, and magnetic resonance imaging (MRI)-based perfusion techniques may assist in identifying patients with elevated intrarenal vascular resistance (31). Moreover, it is important to recognize that for patients with a presumed nephron hypofiltration phenotype, therapies aimed at reducing intraglomerular pressure or reducing afferent perfusion might provide limited benefits or necessitate individualized titration. The therapeutic effects in this scenario may significantly differ from those observed in patients with albuminuric CKD resulting from glomerular hyperfiltration.

Finally, our hypothesis requires validation through predictive mathematical modeling and focused experimental and prospective clinical studies, including early-phase trials of agents that target nephron hypofiltration in nonalbuminuric CKD. An integrated program combining mechanistic and clinical strategies will be essential. Longitudinal evaluations of RRI and renal plasma flow could clarify whether rising intrarenal vascular resistance precedes GFR decline. Advanced imaging, such as arterial spin–labeling MRI and contrast-enhanced ultrasonography, may enable quantitative, *in vivo* assessment of intrarenal perfusion. Ultimately, stratified interventional trials comparing treatment responses in albuminuric versus nonalbuminuric/high-RRI phenotypes will determine the therapeutic significance of this model.
